# Feasibility and Acceptability of Perioperative Application of Biofeedback-Based Virtual Reality vs Control for Pain and Anxiety in Children and Adolescents Undergoing Surgery: Pilot Randomized Controlled Trial

**DOI:** 10.2196/94604

**Published:** 2026-09-16

**Authors:** Anna M Lin, Brenna Rachwal, Lindsey Asti, Zandantsetseg Orgil, Isabela Santiago, Lili Ding, Susmita Kashikar-Zuck, Christopher D King, Sara E Williams, William R Black, Peter C Minneci, Vanessa A Olbrecht

**Affiliations:** 1 Department of Surgery, Nemours Anesthesia and Surgical Outcomes Center, Nemours Children's Hospital, Delaware, Wilmington, DE, United States; 2 Center for Biobehavioral Health, Abigail Wexner Research Institute, Nationwide Children's Hospital, Columbus, OH, United States; 3 Department of Pediatrics, Division of Biostatistics and Epidemiology, Cincinnati Children's Hospital Medical Center, Cincinnati, OH, United States; 4 Department of Pediatrics, Division of Behavioral Medicine and Clinical Psychology, Cincinnati Children's Hospital Medical Center, Cincinnati, OH, United States; 5 Department of Anesthesiology, Perioperative and Pain Medicine, School of Medicine, Stanford Medicine, Stanford, CA, United States; 6 Department of Anesthesiology and Perioperative Medicine, Sidney Kimmel Medical College at Thomas Jefferson University, Nemours Children's Hospital, Delaware, 1600 Rockland Road, Wilmington, DE, 19803, United States, 1 3026680198

**Keywords:** biofeedback, virtual reality, pain, postoperative, pediatrics, pain management, perioperative care

## Abstract

**Background:**

The impact of biofeedback-based virtual reality (VR-BF) on pain and anxiety in pediatric surgical patients remains unexplored.

**Objective:**

The objective of this study was to evaluate the feasibility and acceptability of perioperative VR-BF use in children and adolescents.

**Methods:**

We performed a single-blind, 2-arm, randomized pilot trial. Children aged 12 to 18 years undergoing surgery with expected moderate-to-severe postoperative pain and more than 1 overnight admission were randomized (1:1) to the intervention or control group. Participants in the intervention group received VR-BF using ForeVR (functional outcome response to engaging virtual reality [VR]), a novel, gamified digital intervention that integrates respiratory biofeedback into an immersive VR environment to train children in vagal tone modulation, and the control group used *Manage My Pain*, an app designed to assist patients with recognizing and reflecting on their pain. Participants and caregivers were unblinded to their assigned intervention but blinded to the other arm’s technology; data analysts were masked to treatment allocation. The primary outcome was feasibility, assessed through recruitment, enrollment and randomization, retention, adherence, and outcome data collection. Secondary outcomes evaluated acceptability, including satisfaction and tolerability. Exploratory outcomes, including pain intensity, pain unpleasantness, and anxiety, were assessed in the VR-BF group. This trial was registered at ClinicalTrials.gov (NCT04943874) and is complete.

**Results:**

Of the 202 patients who were eligible for study participation, 70 were enrolled, and 65 (92.9%) patients completed the study (VR-BF, n=30; control, n=35). Female participants accounted for 41 (58.6%) of the participants, and the median age of participants was 16.2 (IQR 14.1-17.5) years. VR-BF participants completed a median of 4 (IQR 3-5) preoperative training sessions and 7 (IQR 2-9) postoperative sessions. Acceptability was high; only 2 (6.7%) would not use VR-BF again. Six VR-BF patients reported mild, transient adverse events (AEs), none of which were serious. VR-BF patients with baseline pain and anxiety scores ≥2 reported a median 1-point (IQR 0-2) reduction in pain intensity, unpleasantness, and anxiety immediately and up to 30 minutes postsession.

**Conclusions:**

Perioperative use of VR-BF is feasible and acceptable in pediatric surgical patients, with high recruitment, retention, and satisfaction and no serious AEs. These findings support a subsequent trial to assess the efficacy and long-term impact of this novel technology.

## Introduction

Managing pediatric postoperative pain remains a critical and persistent challenge [1]. Optimizing pain control is essential for reducing morbidity, promoting recovery, and lowering costs [2]. With growing concerns regarding neurodevelopmental risks and opioid misuse in pediatric populations, clinicians increasingly prioritize opioid-sparing analgesic strategies [3]. Although multimodal approaches have improved postoperative outcomes, optimal postoperative pain management strategies remain elusive.

Nonpharmacological interventions, such as biofeedback (BF) and virtual reality (VR), offer promising strategies for improving perioperative pain management in children [4]. BF can be delivered through multiple modalities [5], but paced breathing interventions that enhance heart rate variability (HRV) promote real-time autonomic regulation and have demonstrated benefits for reducing pain, anxiety, and potentially opioid consumption [6-8]. By increasing vagal activity through slow, diaphragmatic breathing [7,8], these interventions improve autonomic balance and decrease pain perception [6]. Despite their effectiveness, traditional BF approaches face significant implementation challenges, including reliance on trained personnel for administration and difficulty maintaining patient engagement [9-11]. These limitations highlight the need for scalable, engaging approaches that can deliver BF skills to children in real-world settings.

In contrast, VR is widely accessible and provides short-term pain relief through immersive distraction. However, the effects of VR are generally limited to the period of VR exposure [12-16]. Unlike distraction-based VR, which primarily reduces pain by redirecting attention away from painful stimuli, BF teaches patients important self-regulation skills through modification of physiological processes. Delivering BF within an immersive VR environment may further improve motivation, engagement, and adherence to BF by transforming a traditionally repetitive therapy into an interactive, immersive, and rewarding experience [17]. A recent scoping review of BF-based VR (VR-BF) interventions reported improvements in engagement, usability, and patient experience across diverse clinical settings, while additional studies have shown successful training of breathing-based physiological regulation and stress-management skills within VR environments [18,19]. Furthermore, gamification has been shown to improve engagement and motivation among children and adolescents [20]. By combining the physiological benefits of BF with the immersive and engaging nature of VR, VR-BF may function not only as an in-session intervention but also as a tool to teach self-regulation skills that can be applied during periods of stress, pain, and anxiety outside of the VR environment [17,18]. Consistent with this concept, our prior observational work found that many participants reported using the breathing techniques learned during VR-BF sessions when not using ForeVR (functional outcome response to engaging VR), suggesting that these skills are learned and can be applied during recovery [21].

VR-BF has never been implemented in pediatric perioperative care [22]. To address this gap, we conducted a pilot, prospective, randomized controlled trial to evaluate the feasibility and acceptability of perioperative VR-BF use in children. The primary aim was to assess the feasibility of VR-BF use, including recruitment, enrollment and randomization, retention, adherence, and outcome data collection. Secondary outcomes assessed acceptability (satisfaction, tolerability) and exploratory clinical outcomes (pain intensity, pain unpleasantness, anxiety). We hypothesized that VR-BF would be both feasible and well accepted in this population, establishing a foundation for future clinical efficacy trials.

## Methods

### Study Design

This pilot randomized controlled trial used a 2-arm, parallel-group, single-blind design to evaluate the feasibility and acceptability of perioperative VR-BF use in pediatric surgical patients. Participants were randomized to VR-BF (intervention) or *Manage My Pain* (MMP; control). The study was conducted in 2 phases. Phase 1 results have been published [21,23,24], and this manuscript reports phase 2 findings. The study protocol for the phase 2 work was published prior to the enrollment of the first patient [25]. Neither patients nor the public were involved in the design, conduct, or reporting of this trial.

### Ethical Considerations

Phase 2 received institutional review board approval from Nationwide Children’s Hospital (Nationwide; STUDY00002080) on February 24, 2023, and Nemours Children’s Health (NCH), Delaware (IRB#2269307) on January 24, 2025, and was conducted in accordance with ethical research standards. The trial was registered at ClinicalTrials.gov (NCT04943874) on July 8, 2021, prior to phase 1 patient enrollment. Written informed consent was obtained from all participants, or from a parent or legal surrogate for minors, with assent from those older than 11 years. The trial adhered to CONSORT (Consolidated Standards of Reporting Trials) guidelines (Checklist 1) [26].

### Data Handling

Data were collected and managed using the REDCap tools hosted at both institutions. REDCap is a secure, web-based software platform designed to support data capture for research studies [27].

### Patient Population

Seventy children and adolescents undergoing surgeries with expected moderate-to-severe postoperative pain requiring management by the Acute Pain Service and more than 1 overnight admission were enrolled at Nationwide and NCH over 2 years (March 27, 2023, to July 22, 2025). Participants were enrolled prospectively before surgery and randomized after consent procedures were completed. The last participant was enrolled on July 22, 2025, and the study’s primary completion date was August 22, 2025.

### Inclusion and Exclusion Criteria

Participants were eligible to participate if they were aged 12 to 18 years; were fluent in English; were undergoing surgery necessitating an overnight postoperative admission; were being managed by the Acute Pain Service; and had access to a mobile device or computer for study participation. Participants randomized to the VR-BF group were provided with all required equipment by the study team prior to their first study visit, including a ForeVR device and a VR headset. Exclusion criteria included non-English speakers; a history of developmental delay, psychiatric conditions associated with hallucinations or delusions; epilepsy or a seizure disorder; significant motion sickness; chronic pain; or conditions preventing VR headset use (eg, craniofacial abnormalities and head or neck surgeries).

### VR-BF (Intervention)

**Figure 1.**
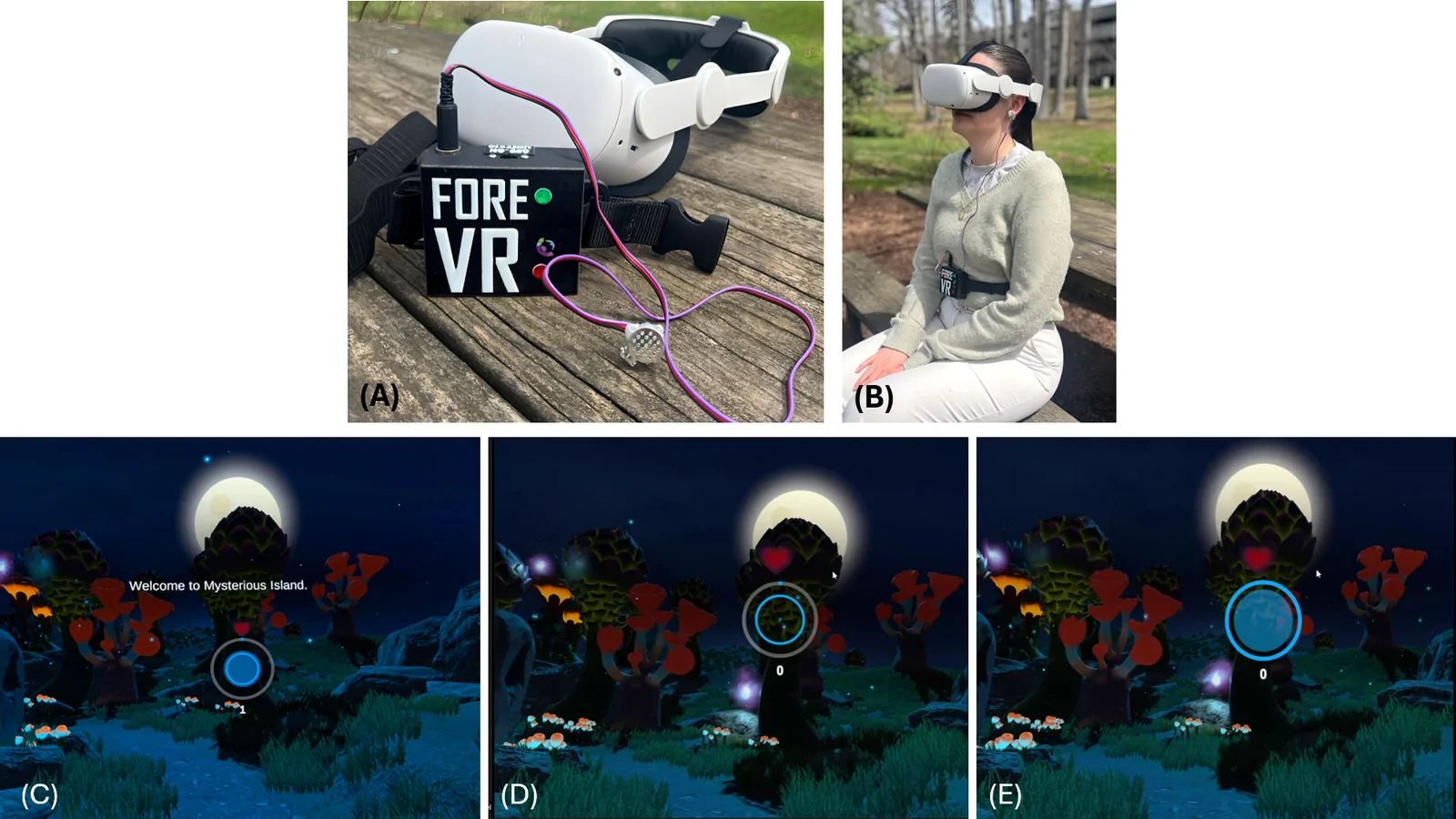
ForeVR (functional outcome response to engaging virtual reality [VR]) device and *Mysterious Island* game. (A) ForeVR consists of Bluetooth-integrated hardware that provides real-time measures of heart and respiratory rate and software that integrates the physiological data stream into the VR experience; (B) a research team member using ForeVR; (C) the opening screen for the *Mysterious Island* game, which welcomes the user to the island and provides user instructions; (D) an unfilled blue circle that serves as a breathing prompt; (E) a solid blue circle that displays the patient’s inhale and exhale in real time, allowing instant feedback. The heart in the frame beats in sync with the participant’s heart rate and verifies connectivity between ForeVR and the headset.

ForeVR is a novel, noncommercial, custom-built, VR-BF platform developed by the study team. The device used in this study was the first-generation prototype, and this represents its first use in a research setting. ForeVR integrates real-time patient physiological data into a gamified VR environment, guiding patients to enhance vagal tone through slowing their breathing and increasing HRV [6,28]. Patients’ breathing controls in-game actions, providing real-time feedback that reinforces targeted breathing patterns while continuously capturing respiratory and cardiac data to assess the physiological impact of VR-BF. ForeVR connects to the Meta Quest VR headset (Meta Platforms) via Bluetooth. In the proprietary *Mysterious Island* game, patients navigate the virtual environment by synchronizing their breathing with a visual prompt that also provides immediate visual feedback on their breathing. Successful synchronization advances gameplay and unlocks rewards to enhance engagement (Figure 1).

### MMP (Control)

MMP is a commercially available pain management app designed to help patients recognize, document, and reflect on their pain experiences. It was selected as an active digital control because it provides engagement with a health-related technology without incorporating the core therapeutic components of the intervention, including BF, immersive VR, or physiologic self-regulation. This selection allowed the evaluation of VR-BF while minimizing confounding related to technology use alone and isolating the potential contribution of VR-based physiologic self-regulation beyond standard digital engagement. In a systematic evaluation of commercially available pain apps, MMP was found to be appropriate for adolescents and scored highly on engagement (4.2/5), functionality (4.5/5), and overall quality (4.06/5) [29]. This approach is consistent with recommendations for early-phase digital health trials, where active comparators can help isolate intervention-specific effects from nonspecific benefits associated with technology use and digital engagement [25,29].

### Randomization and Masking

Participants were randomized in a 1:1 ratio into the study arms using a secure, web-based randomization tool [30]. The randomization sequence was generated prior to study initiation, with allocation determined by participant number. Participants were masked to study arm allocation and informed only that they were participating in a study evaluating technology-based interventions for perioperative recovery, without disclosure of the specific technologies used in each study arm. During consent and enrollment, participants were told that they would be assigned to 1 of the 2 study interventions but were not informed of the study hypotheses regarding potential benefits of either intervention. Following randomization, participants received information and training only for their assigned technology. Investigators enrolling participants and care providers could not be masked due to the nature of the study, but outcome assessors and data analysts were masked to the treatment allocation.

### Conduct of the Study

Potential patients were identified using a custom EPIC-generated screening report by the principal investigator (VAO). Research staff approached candidates and scheduled a consent visit for eligible patients expressing interest. Enrollment occurred following written informed consent and assent. Patient demographic characteristics, including sex, race, and ethnicity, were collected from the electronic medical record and entered based on patient self-report.

### Preoperative Protocol

Before surgery, all participants viewed a standardized video outlining the potential benefits of the technology. Participants randomized to VR-BF were provided with a Meta Quest headset and a ForeVR device by the study team and received standardized training in device setup and use. They retained this equipment throughout study participation, allowing continued use during hospitalization and after discharge if they left the hospital prior to completion of the 7-day postoperative intervention period. Control group participants received training on the MMP app. Both groups were instructed to complete daily 10-minute training sessions for 5 days before surgery, documenting each session through completion of a diary entry. For consistency, each use of the assigned technology was defined as a study session. During the preoperative period, sessions were referred to as training sessions because they were intended to familiarize participants with the technology and promote skill acquisition prior to surgery. Participants were also asked to complete a diary entry following each session to document intervention use and study-related outcomes.

### Postoperative Protocol

After surgery, participants continued their assigned intervention for 7 days and were asked to complete three 10-minute sessions (morning, noon, and evening) daily, spaced approximately 8 hours apart. This dosing schedule was selected to provide repeated opportunities for patients to practice and reinforce breathing-based self-regulation skills during the period of greatest postoperative pain burden, and was informed by our preliminary work and established BF training approaches that emphasize frequent and repeated practice to facilitate skill acquisition and autonomic regulation [6,7,23,25]. Patients discharged before day 7 were instructed to complete the protocol at home. Participants in the VR-BF group retained the study-provided Meta Quest headset and ForeVR device until study completion, allowing for continued home use. Participants returned all study equipment at the time of study completion. Control participants submitted app-generated usage data and session-specific diary entries, and VR-BF participants completed diary entries with each session.

### Study Measurements

Consistent with the pilot nature of this study, outcomes included 3 domains: feasibility (primary outcome), acceptability (secondary outcome), and exploratory clinical outcomes in the intervention group. Feasibility outcomes included recruitment (patients screened and assessed for eligibility), enrollment and randomization (consent and acceptance of randomization), retention (completion of the final visit and questionnaire), adherence (preoperative: completion of more than 1 of 5 training sessions; postoperative: completion of more than 1 session per day for 7 days), and outcome data collection (pain, anxiety, and opioid use). Acceptability outcomes included satisfaction and tolerability. Feedback from patients and caregivers was collected using study-specific questionnaires with a 5-point Likert scale at the final study visit. Tolerability was defined by the incidence of adverse events (AEs) and serious AEs, defined as any untoward medical or psychological occurrence during participation that substantially disrupted participant well-being [31]. Because no validated instruments were available to assess the acceptability and feasibility of a novel perioperative VR-BF intervention in pediatric surgical patients, study-specific questionnaires were developed for this pilot study using items adapted from established implementation science measures, including the feasibility of intervention measure and theory-informed acceptability frameworks [32-34]. These questionnaires were intended to descriptively assess participant and caregiver perceptions and were not used for hypothesis testing or efficacy evaluation.

Clinical outcomes included pain intensity, pain unpleasantness, and anxiety, measured using the Numeric Rating Scale (NRS, 0‐10) [35]. These scores were collected during postoperative sessions only. Participants completed baseline ratings immediately before the session, immediately after session completion, and again at 15 and 30 minutes following session completion. These assessments were repeated for each postoperative session. A standardized script clarified pain types using an analogy—intensity as “volume of music” and unpleasantness as “dislike of the music”—and was used to familiarize participants with the 2 types of reported pain [36]. Opioid consumption was extracted from the electronic medical record during hospitalization, supplemented by patient diary entries after discharge. These measures were collected for feasibility purposes and will serve as key clinical efficacy endpoints in future trials. Exploratory analyses were limited to within-session changes observed during VR-BF use and were intended to characterize the direction and magnitude of response associated with the intervention rather than to compare treatment groups.

### Sample Size

For this pilot feasibility and acceptability study, no confirmatory hypothesis testing was conducted. The sample size is based on the estimation of 2-sided CIs for a single proportion using the exact formula of the binomial probabilities. Estimating a 95% CI with a targeted width of 0.22 and an assumed proportion of 80% requires a sample size of 70 (35 per group), resulting in 56 patients (28 per group) included in the analysis after 80% retention [25].

### Statistical Analysis

Continuous variables were reported as median values with IQRs, while categorical variables were reported as counts and percentages. Descriptive statistics were generated using SAS Enterprise Guide 8.4 (SAS Institute). Missing values were excluded except for demographics, for which missingness was represented as an independent level and presented as such in Table 1.

**Table 1.** Patient demographics and characteristics.

Characteristics	Overall study population	VR-BF^a^ group	Control group
Overall, n (%)	70 (100)	31 (44.3)	39 (55.7)
Surgery type, n (%)			
Orthopedic	51 (72.9)	24 (77.4)	27 (69.2)
Abdominal	9 (12.9)	3 (9.7)	6 (15.4)
Chest	4 (5.7)	1 (3.2)	3 (7.7)
Colorectal	1 (1.4)	1 (3.2)	0 (0)
Urology	1 (1.4)	1 (3.2)	0 (0)
Other	3 (4.3)	0 (0)	3 (7.7)
Missing	1 (1.4)	1 (3.2)	0 (0)
Sex, n (%)			
Male	29 (41.4)	8 (25.8)	21 (53.8)
Female	41 (58.6)	23 (74.2)	18 (46.2)
Race, n (%)			
White	56 (80)	26 (83.9)	30 (76.9)
Black	7 (10)	3 (9.7)	4 (10.3)
American Indian/Alaska Native	1 (1.4)	0 (0)	1 (2.6)
Asian	1 (1.4)	0 (0)	1 (2.6)
Biracial	1 (1.4)	0 (0)	1 (2.6)
Other	4 (5.7)	2 (6.5)	2 (5.1)
Ethnicity, n (%)			
Hispanic	4 (5.7)	2 (6.5)	2 (5.1)
Non-Hispanic	65 (92.9)	29 (93.5)	36 (92.3)
Unknown	1 (1.4)	0 (0)	1 (2.6)
Weight (kg), median (IQR)	61.4 (50.4‐74.7)	57.8 (49.0‐71.6)	62.9 (50.7‐85.5)
Height (inches), median (IQR)	163.5 (158.9‐172.5)	161.0 (158.9‐165.0)	165.7 (158.0‐173.6)
Hospital LOS^b^ (days), median (IQR)	3 (3-4)	3 (3-4)	3 (3-5)
ASA^c^ physical status, n (%)			
1	8 (11.6)	6 (20)	2 (5.1)
2	43 (62.3)	18 (60)	25 (64.1)
3	16 (23.2)	4 (13.3)	12 (30.8)
4	2 (2.9)	2 (6.7)	0 (0)

a VR-BF: biofeedback-based virtual reality.

b LOS: length of stay.

c ASA: American Society of Anesthesiologists.

## Results

### Patient Characteristics

Seventy patients were enrolled (31 VR-BF, 39 control; Table 1), with a median age of 16.2 (14.1-17.5) years. Female participants comprised 74.2% (23/31) and 46.2% (18/39) of the VR-BF and control groups, respectively. Most participants had an American Society of Anesthesiologists (ASA) physical status of 1 or 2 (51/70, 73.9%). Surgical procedures were predominantly orthopedic (51/70, 73.9%), followed by abdominal (9/70, 13.0%), chest (4/70, 5.8%), colorectal (1/70, 1.5%), and urologic (1/70, 1.5%). The median hospital stay was 3 (IQR 3-4) days.

### Feasibility

**Figure 2.**
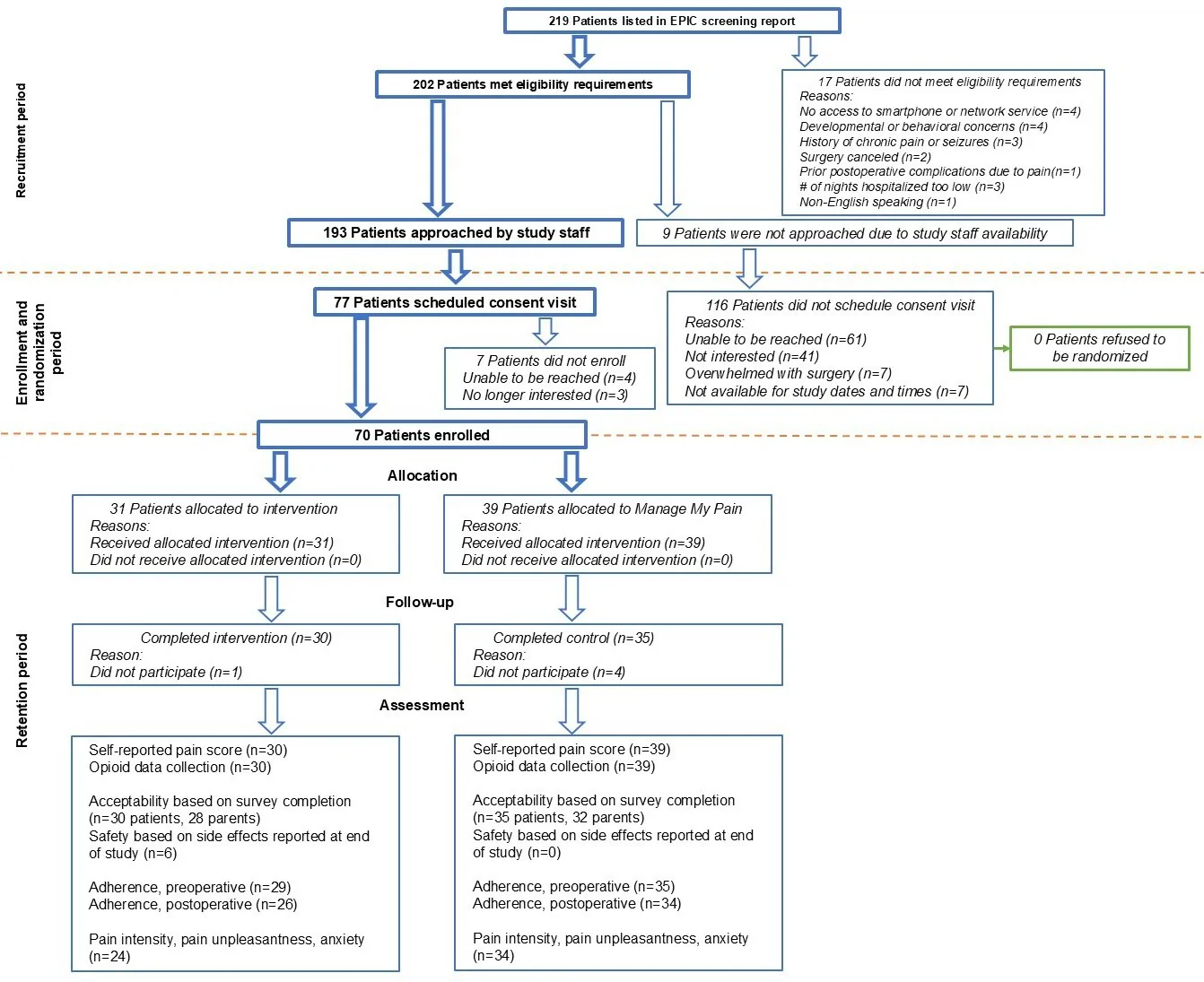
CONSORT (Consolidated Standards of Reporting Trials) diagram depicting participant flow, including details of recruitment, enrollment, randomization, retention, and follow-up. Self-reported pain scores and opioid data were reported for participants who underwent surgery and had data collected. Adherence was defined by completion of at least 1 study activity. Pain intensity, pain unpleasantness, and anxiety data were reported for participants who used their assigned technology and reported pain or anxiety scores at least once during the study period. VR-BF: biofeedback-based virtual reality.

Of the 219 patients screened for eligibility, 202 (92.2%) met eligibility criteria. Of 202 patients, 193 (95.5%) were approached for participation and 70 enrolled, with no refusals for randomization (Figure 2). The most common reasons for not scheduling a consent visit were inability to reach the patient (n=61) and participants’ loss of interest in study participation (n=41). Sixty-five (92.9%) participants completed the study; 5 (7.1%) were lost to follow-up or withdrew after enrollment. Outcome data capture was 97.2% (n=68) for opioid use and pain scores at Nationwide and 100% for both outcome measures at NCH.

Preoperatively, 91.4% (64/70) of participants completed more than 1 assigned session and the corresponding diary entry. In the VR-BF group, the median number of days with completed training sessions was 4 (IQR 3-5), with a median of 5 (IQR 3-5) completed diary entries. The control group showed similar adherence, completing a median of 5 (IQR 4-5) sessions and 5 (IQR 4-5) diary entries.

**Figure 3.**
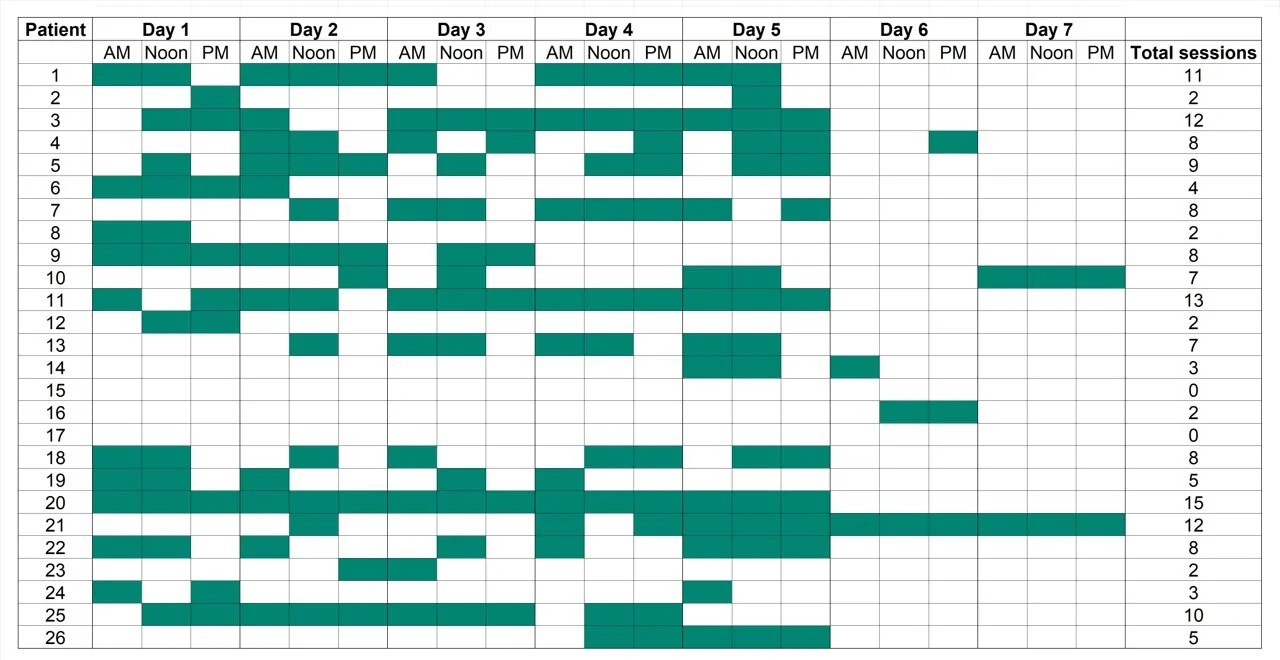
Tile diagram showing ForeVR (functional outcome response to engaging virtual reality) device usage among the 26 biofeedback-based virtual reality (VR-BF) participants in the postoperative study period. There were 21 possible sessions in the morning (AM), noon (midday), and evening (PM) for the 7 days of the postoperative period. A green tile indicates use of the device during a potential session, while a blank tile indicates no use. The total number of sessions is displayed in the rightmost column labeled “Total sessions.” The maximum number of sessions was 21, and the minimum was 0. Only participants completing at least 1 diary entry after surgery are represented in this diagram.

Postoperatively, 85.7% (60/70) of participants completed more than 1 session and diary entry (Figure 2). Among VR-BF participants, the median number of completed sessions was 7 (IQR 2-9), with 12.5 (IQR 5.0-16.0) diary entries, and a maximum of 15 sessions and 18 diary entries (Figure 3). VR-BF was used for a median of 4 (IQR 2-5) days, with a median of 2 (IQR 1.6-2.4) sessions per day. In the control group, the median number of completed sessions was 8 (IQR 5-13), with 11.5 (IQR 6.0-15.0) diary entries, and a maximum of 15 sessions and 18 diary entries. The MMP app was used for a median of 5 (IQR 4-5) days, with a median of 2 (IQR 1.6-2.6) sessions per day.

Several participants experienced technical difficulties with the first-generation ForeVR prototype during their study participation, including hardware and connectivity issues that occasionally interrupted session completion and contributed to missed sessions. These issues are reflected in the adherence data and informed subsequent device refinements.

### Acceptability

**Figure 4.**
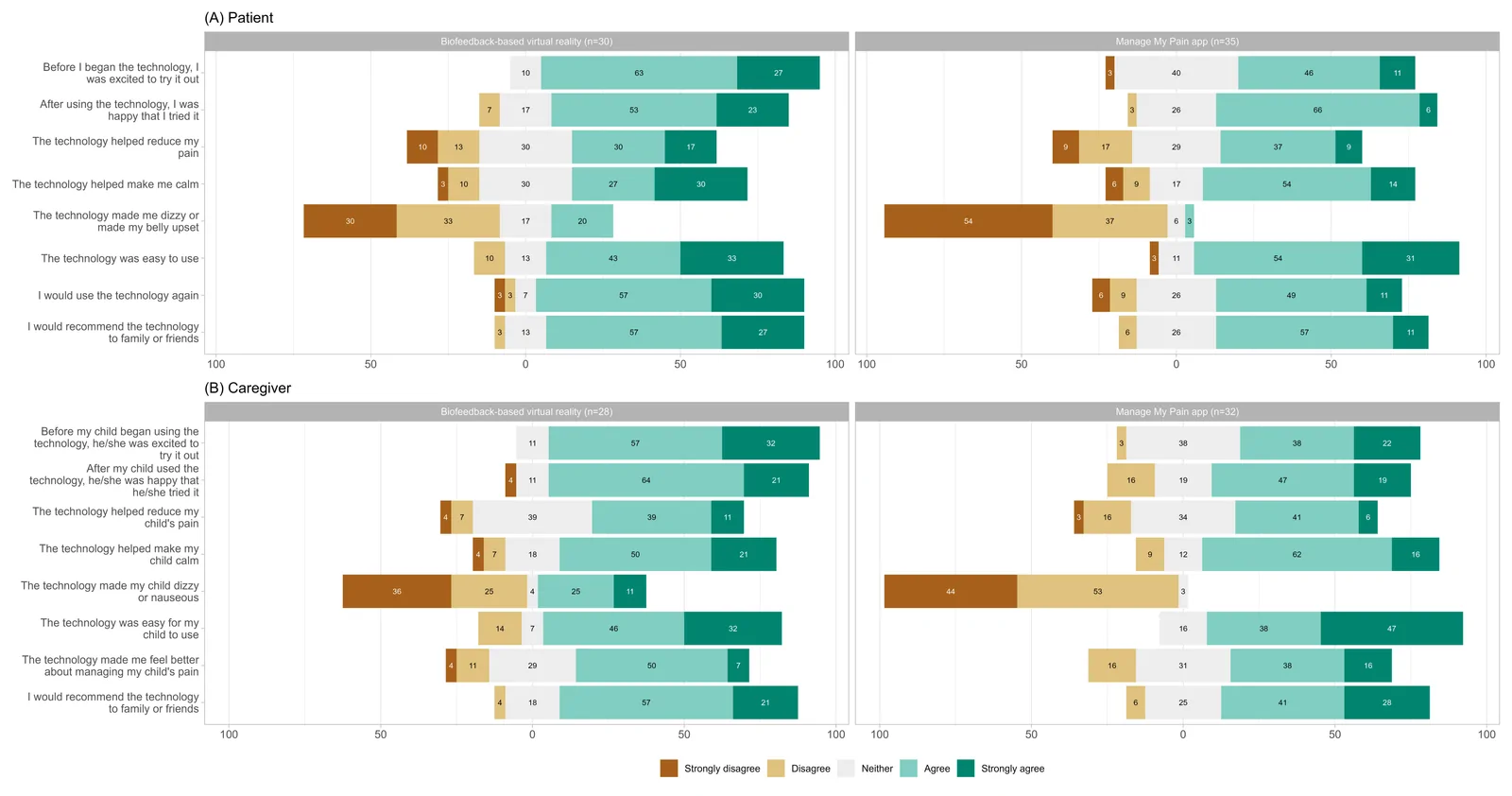
Stacked bar plot of (A) patient and (B) caregiver responses to selected survey items gauging their perceptions of biofeedback-based virtual reality using ForeVR and the *Manage My Pain* app. The vertical reference line indicates the response of “neither agree nor disagree.” The overall length of the bar corresponds to the number of patients completing each question, and the numbers in the bar represent the proportion of responses corresponding to each selected Likert-scale choice. ForeVR: functional outcome response to engaging virtual reality.

Acceptability was assessed at the final study visit using study-specific questionnaires completed by both participants and caregivers. Those answering the questionnaires were asked to reflect on their perceptions of the assigned technology, including their level of excitement prior to initiating use and their satisfaction after study completion. Satisfaction was high for both groups (Figure 4). When reflecting on their perceptions prior to study participation, 90.0% (27/30) of patients and 89.3% (25/28) of parents expressed excitement about using ForeVR, compared to 57.1% (20/35) of patients and 59.4% (19/32) of parents for MMP. After participation, 76.7% (23/30) of patients and 85.7% (24/28) of parents were pleased with ForeVR, while 71.4% (25/35) and 65.6% (21/32) were satisfied with MMP. Nearly half (14/30, 46.7%) of participants agreed or strongly agreed that VR-BF reduced pain, and 56.7% (17/30) agreed that it promoted calmness. In the control group, 45.7% (16/35) agreed or strongly agreed that MMP helped to reduce pain, and 68.7% (24/35) agreed it promoted calmness. Parents of participants in both groups echoed these positive perceptions.

Postoperatively, patients completed diary entries at each session; each entry asked about side effects potentially related to device use (nausea, vomiting, dizziness, headache, other, or none). Nine of the 60 participants reported at least one AE in these diaries; all were in the VR-BF group [13]. At the final study visit, 6 VR-BF participants self-reported mild, transient AEs directly to a study team member; no serious AEs occurred. Detailed side effects reported during this visit included 2 participants with headache (possibly intervention-related); 1 with headache, backache, and stomachache (possibly intervention-related); 1 with headache (intervention-unrelated); 1 with nausea (intervention-related); and 1 with headache, nausea, and dizziness (intervention-related). These side effects are known and consistent with those commonly reported during VR use [13].

### Exploratory Observations of Outcomes

**Figure 5.**
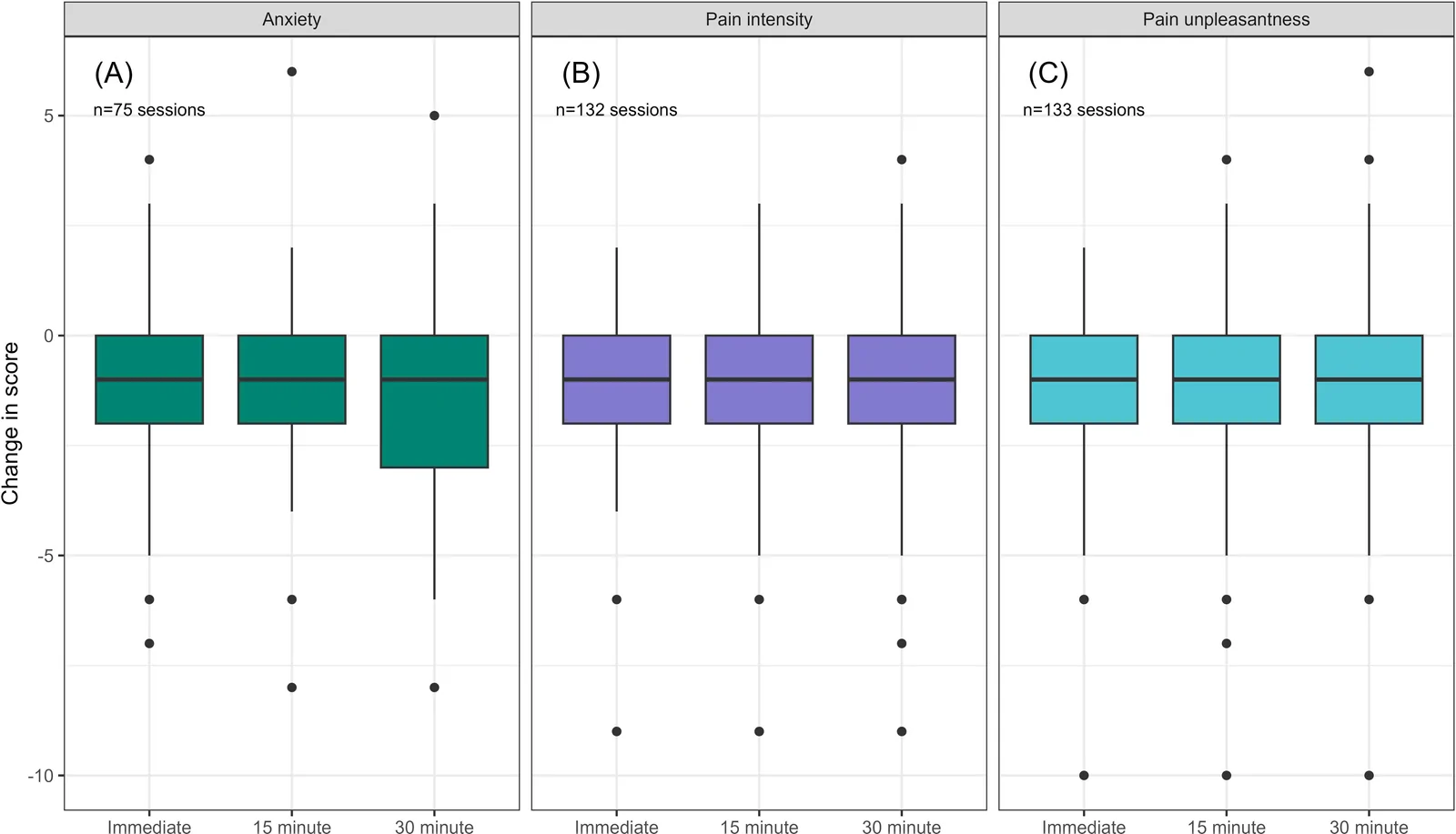
Box-and-whisker plots showing the distribution of changes in (A) anxiety, (B) pain intensity, and (C) pain unpleasantness following biofeedback-based virtual reality (VR-BF) sessions with a starting pain or anxiety score of >2. Boxes represent the IQR (25th-75th percentile), with the center line indicating the median (50th percentile). Whiskers show the full range of values, and individual dots denote outliers. The maximum possible value was 8, and the minimum possible value was −10. The maximum observed score was 6, and the minimum observed score was −10. Negative values indicate improvement in the pain or anxiety score. The horizontal red dotted line at 0 was included to improve the readability of positive vs negative values.

Consistent with the exploratory objectives of this pilot study, analyses were limited to within-session changes observed during VR-BF use among participants with presession pain or anxiety scores ≥2, as lower scores indicated adequate pain control and limited potential for improvement. Among 546 observations in this subgroup, VR-BF participants demonstrated a median 1-point (IQR 0-2) reduction on the NRS for both pain intensity and unpleasantness immediately postsession, with improvements persisting at 15 and 30 minutes following session completion (Figure 5). The maximum reductions reported were 9 points for pain intensity and 10 points for pain unpleasantness immediately after VR-BF, and these changes were sustained at 30 minutes. A 1-point decrease is considered clinically meaningful for postoperative pain reduction [37]. Anxiety showed a similar pattern, with a median 1-point decrease (IQR 0-2) immediately and at 15 minutes, and a 1-point decrease (IQR 0-3) at 30 minutes. Maximum anxiety reductions were 7 points immediately following session completion, and 8 points at 15 and 30 minutes postsession.

## Discussion

This pilot trial is the first to evaluate a perioperative VR-BF intervention in a pediatric surgical population and the first to use ForeVR, a novel, first-generation prototype developed by our team in a research setting. Establishing feasibility and acceptability of this technology in children and adolescents undergoing surgery is an essential first step toward introducing VR-BF into pediatric perioperative care, a domain where no prior evidence exists and where adult research is also extremely limited [38,39]. These results lay the groundwork for future studies to refine intervention design, rigorously assess efficacy, and explore the potential of VR-BF to improve perioperative outcomes in children.

Our findings demonstrate strong feasibility and acceptability of this intervention. Of 202 patients screened, 70 were enrolled, with excellent retention (65/70, 92.9%) and adherence in both the preoperative (64/70, 91.4% completing more than 1 session) and postoperative phases (60/70, 85.7% completing more than 1 session). Participants engaged pragmatically, completing a median of 2 sessions per day over a median of 4 to 5 postoperative days, with only mild, transient side effects reported in the VR-BF group, consistent with known VR-related side effects [13]. Satisfaction was high among participants and caregivers in both study groups, reinforcing acceptability. Exploratory analyses in the intervention group demonstrated a median 1-point reduction in pain intensity, pain unpleasantness, and anxiety following VR-BF sessions among participants with baseline symptoms greater than 2, with similar reductions observed at 15- and 30-minute follow-up assessments. Although this magnitude of change is considered clinically meaningful for postoperative pain reduction [37], these findings should be interpreted as hypothesis-generating given that this study was designed as a feasibility and acceptability trial rather than an efficacy trial.

Evidence for VR use in pediatric perioperative pain and anxiety management remains limited, with most studies focused on distraction-based and guided relaxation–based VR for preoperative anxiety or procedural pain reduction [12,40]. Our prior pilot work showed transient benefits of distraction-based and guided relaxation–based VR for postoperative pain but was limited by a single-session design and the lack of a comparator, leaving questions about sustained benefit, causality, and true efficacy unanswered [41,42]. This study advances the field by introducing VR-BF, an approach that integrates physiologically grounded BF therapy with immersive VR engagement, promoting autonomic regulation through slow, resonance-frequency breathing and enhancing HRV and vagal tone to reduce pain [6-9,28,43-46]. Engagement is also challenging, as patients often describe traditional BF as “extremely boring,” leading to reduced engagement [18,47]. VR-based delivery addresses these barriers by gamifying BF, improving usability, and enhancing scalability [20]. The ForeVR platform leverages these principles to enable independent HRV optimization in an immersive, gamified environment.

Several insights from this work will inform future trials. Three daily postoperative sessions were selected to provide repeated opportunities for skill acquisition and reinforcement during recovery, consistent with established BF training approaches and our preliminary work [6,7,23,25]. However, participant feedback and adherence data show that this schedule was overly burdensome for patients managing multiple postoperative demands. Therefore, future studies will adopt a more pragmatic twice-daily dosing schedule with flexible timing and open diary windows to better align with real-world postoperative routines and improve participant adherence. Surgeon endorsement and parental engagement were critical for adherence, highlighting the importance of care team involvement. Patients referred to study participation by their surgeon did especially well with adherence, underscoring our plan to engage surgeons directly in the recruitment and enrollment process. Technical issues associated with the first-generation ForeVR prototype also impacted adherence. During the study, challenges included hardware reliability, occasional connectivity disruptions between ForeVR and the Meta Quest headset, and physiologic data collection limitations that interfered with session participation and/or completion while also impacting the user experience. Identification of these issues was an important outcome of this study, as these issues have directly informed design enhancements and workflow improvements. Since study completion, substantial modifications have been implemented to enhance device reliability, improve the user experience, and strengthen physiologic data capture and integration.

Because this trial was designed as a feasibility and acceptability study, clinical outcomes were evaluated on an exploratory basis and should be interpreted only as hypothesis-generating. Exploratory analyses demonstrated associations between VR-BF use and changes in pain and anxiety among participants with baseline symptoms greater than 2. While these findings do not permit conclusions regarding efficacy, they support further evaluation of VR-BF in fully powered clinical trials. Prior studies suggest that VR interventions may have greater analgesic effects in settings associated with higher baseline pain severity and procedural pain [48,49]. Consistent with these observations, future studies will focus on more homogeneous surgical populations with high postoperative pain burden.

A strength of this study was the use of an active digital comparator rather than usual care alone. By selecting a technology-based intervention that encouraged symptom monitoring and engagement but did not include BF, breathing training, immersive VR, or physiologic self-regulation, the study design helped distinguish the feasibility and acceptability of VR-BF from the effects of digital engagement alone. This approach allowed for a more rigorous assessment of intervention-specific factors associated with VR-BF while minimizing confounding related to technology use itself.

This study has several limitations. As a pilot feasibility and acceptability trial, this study was not designed or powered to evaluate efficacy or detect differences in clinical outcomes between groups. Inclusion of a heterogeneous surgical population increased the generalizability of study findings and allowed evaluation of implementation across diverse perioperative settings; however, variability in procedure type, pain burden, and recovery trajectories limited comparability across participants. In addition, the postoperative intervention schedule required up to 3 sessions per day for 7 days, which some participants perceived as burdensome and may have contributed to reduced adherence. Because one of the goals of this study was to evaluate acceptability of the intervention schedule, these findings provide important information regarding study burden and will directly inform protocol refinements for future trials. Future studies will focus on more homogeneous surgical populations with standardized pain management pathways and a more pragmatic intervention schedule.

Technical challenges associated with the first-generation ForeVR prototype contributed to missed sessions, reduced adherence, and lower satisfaction among some participants. Although these issues are common during early-stage technology development, they may have influenced feasibility and acceptability metrics.

Acceptability measures were collected at study completion and relied on participants’ and caregivers’ recall, including retrospective assessment of excitement and expectations prior to technology use. As a result, responses may have been influenced by recall and expectation effects. In addition, participants willing to enroll in a technology-based intervention study may have been more receptive to digital health approaches than the broader surgical population, potentially resulting in overestimation of acceptability. Finally, although participant and caregiver feedback informed intervention refinement, patients and families were not involved as research partners in the design, conduct, and reporting of the study. Future work will incorporate patient and family stakeholders to further optimize intervention implementation and evaluation.

Despite these limitations, this study successfully met its primary and secondary objectives, confirming the feasibility and acceptability of perioperative VR-BF use and generating actionable insights for protocol refinement, device enhancements, and engagement strategies. These findings establish a robust platform for future research and highlight VR-BF’s promise as an innovative, scalable, and patient-centered adjunct to multimodal perioperative care.

In conclusion, this pilot study demonstrated that perioperative VR-BF use is feasible and acceptable in pediatric surgical patients, with high recruitment, retention, and participant satisfaction and no serious AEs. The study also identified important refinements to the intervention, technology, and study procedures that will inform future trial design and implementation. While exploratory observations suggested reductions in pain and anxiety following VR-BF sessions, the study was not designed or powered to evaluate efficacy, and these findings primarily serve to inform future investigation. Collectively, these results support further investigation of VR-BF in a fully powered clinical trial and highlight its promise as a scalable, patient-centered approach for delivering BF in perioperative care.

## Supplementary material

10.2196/94604 Checklist 1 CONSORT checklist.

## Abbreviations

AE: adverse event
BR: biofeedback
CONSORT: Consolidated Standards of Reporting Trials
ForeVR: functional outcome response to engaging virtual reality
HRV: heart rate variability
MMP: Manage My Pain
Nationwide: Nationwide Children’s Hospital
NCH: Nemours Children’s Health
NRS: Numeric Rating Scale
VR: virtual reality
VR-BF: biofeedback-based virtual reality
